# Zona Pellucida Protein 2 (ZP2) Is Expressed in Colon Cancer and Promotes Cell Proliferation

**DOI:** 10.3390/cancers13081759

**Published:** 2021-04-07

**Authors:** Dominik Kraus, Alexander Glassmann, Carsten Golletz, Glen Kristiansen, Jochen Winter, Rainer Probstmeier

**Affiliations:** 1 Department of Prosthodontics, Preclinical Education and Material Sciences, University of Bonn, Welschnonnenstr 17, 53111 Bonn, Germany; dominik.kraus@ukbonn.de; 2 Life Science Inkubator, Ludwig-Erhard-Allee 2, 53175 Bonn, Germany; alexander.glassmann@h-brs.de; 3 Institute of Pathology, Venusberg-Campus 1, University Hospital of Bonn, 53127 Bonn, Germany; carsten.golletz@ukbonn.de (C.G.); glen.kristiansen@ukbonn.de (G.K.); 4 Oral Cell Biology Group, Department of Periodontology, Operative and Preventive Dentistry, University of Bonn, Welschnonnenstr. 17, 53111 Bonn, Germany; 5 Neuro- and Tumor Cell Biology Group, Department of Nuclear Medicine, Venusberg-Campus 1, University Hospital of Bonn, 53127 Bonn, Germany; r.probstmeier@gmx.net

**Keywords:** molecular pathology, neoexpression, tumor diagnosis, cancer biomarker, zona pellucida protein 2 ZP2

## Abstract

**Simple Summary:**

Our study shows ZP2 to be a new biomarker for diagnosis, best used in combination with other low abundant genes in colon cancer. Furthermore, ZP2 promotes cell proliferation via the ERK1/2-cyclinD1-signaling pathway. We demonstrate that ZP2 mRNA is expressed in a low-abundant manner with high specificity in subsets of cancer cell lines representing different cancer subtypes and also in a significant proportion of primary colon cancers. The potential benefit of ZP2 as a biomarker is discussed. In the second part of our study, the function of ZP2 in cancerogenesis has been analyzed. Since ZP2 shows an enhanced transcript level in colon cancer cells, siRNA experiments have been performed to verify the potential role of ZP2 in cell proliferation. Based on these data, ZP2 might serve as a new target molecule for cancer diagnosis and treatment in respective cancer types such as colon cancer.

**Abstract:**

Background: Zona pellucida protein ZP2 has been identified as a new colon tumor biomarker. Its transcripts were specifically expressed in four out of four human colon cancer cell lines and enhanced in about 60% of primary colon cancer tissues when compared to matched healthy ones. ZP2 down-regulation by siRNA led to a decreased proliferation rate, EXOSC5 transcript, cyclin D1 protein level, and ERK1/2 phosphorylation state. Methods: Sensitivity and quantitative expression analysis of ZP2 transcripts in tumor and matched normal colon tissue was performed with respective cDNA preparations. Silencing RNA effects on colon cancer cells were examined by q-PCR, western blot, and proliferation rate experiments. Results: In a significant portion of 69 primary colon tumor samples, the molecule showed a low but specific expression, which revealed a sensitivity value of around 90% and a specificity value of 30% when matched to the respective normal counterparts. Down-regulation of ZP2 protein by siRNA led to a decreased proliferation rate, EXOSC5 and cyclin D1 level, and phosphorylation state of ERK1/2. ZP2 has also been found to be a cell membrane-bound protein. Conclusion: ZP2 shows an enhanced expression level in colon cancer tissue and, thus, can be used as a diagnostic tool, albeit in combination with other biomarkers. Its character as a membrane protein makes ZP2 even a potential target molecule for tumor therapy, especially as it positively affects colon cancer cell proliferation.

## 1. Introduction

The application of available tumor markers is rather limited, mainly because of the fact that such markers are also expressed by normal cells, albeit often to a lower extent, and, thus, do not allow a simple yes/no decision for tumor diagnosis. Tumor initiation and development are accompanied by alterations of the genetic material, comprising DNA mutations and epigenetic modifications. Regarding the DNA sequence proper, this can lead to a high degree of random and stable mutations, especially in cells with a so-called mutator phenotype that is generated, for example, via the presence of error-prone DNA polymerases [1]. Additionally, the epigenome is substantially remodeled in the context of oncogenesis and characterized by open and, in consequence, highly accessible chromatin [2,3]. The mutator phenotype as well as the presence of highly accessible chromatin adds to an increase in stochastic gene expression, a phenomenon that, in principle, is present also under non-pathological conditions [4,5]. In bacterial cells, it has been demonstrated that the actual amount and amplitude of stochastic gene expression, termed noisiness, is dependent on the specific genetic background [6]. A comparison of deterministic vs. stochastic simulations of cellular gene expression shows that under certain circumstances (high numbers of mRNA molecules, large cell volume) intracellular gene expression rates are comparable, whereas otherwise (low numbers of mRNA molecules, small cell volume) high fluctuations are present [4]. For the expression of low abundant genes, an extrapolation of such a scenario from single cells to cell populations makes it likely that (1) in larger cell populations, higher fluctuation rates will provoke an overall increase in the threshold of detection (in our case RT-PCR), which is dependent on the population size, and (2) a reduction of the population size will lower the probability of detecting an expression of the low abundant gene at all, but, when present, will increase the probability of a higher expression level (in comparison to the one in larger cell populations).

In principle, via such deregulations in cancer cells, the expression of each gene could be affected and, in particular, could lead to gene neoexpression. An experimental support of this assumption is provided by recent data of ours demonstrating that the matrix metalloproteinase MMP20, whose expression in normal development is restricted to defined populations of ameloblasts and odontoblasts, is detectable in main human tumor subgroups [7].

We have now extended these types of studies to the zona pellucida protein ZP2. The zona pellucida (ZP) is a porous matrix-like structure that surrounds the oocyte and, in humans, consists of four glycoproteins designated as ZP1, ZP2, ZP3, and ZP4. Whereas ZP2 and ZP3 are expressed by oocytes of all mammalian species so far investigated, in some species, ZP1 or ZP4 can be missing. During the fertilization process, capacitated sperm cells interact with the ZP, whereby in humans all four ZP glycoproteins participate in a more or less dominant fashion. Like the other human ZP proteins, ZP2 is also a secreted glycosylated protein that consists of 745 amino acids and harbors the so-called ZP-domain, comprising about 260 amino acids, and is most likely important for ZP assembly [8,9]. In the (mouse) oocyte, ZP2 is first synthesized as a transmembrane-protein, which has to be cleaved after binding acrosomal proteins before its ectodomain is integrated into the zona pellucida matrix [10]. To our knowledge, ZP2-specific transcripts and proteins have so far only been detected in oocytes of various species and prostate cancer cells [11]. We have chosen ZP2 for further investigations regarding its potential as a new target molecule in colon cancer diagnosis/treatment. Recent studies with tumor cell lines showed ZP2 expression in few cell lines, whereas ZP3 has been found in various different tumors [12,13]. Noteworthily, ZP3 has already been found in human colorectal cancer [14] and also in human and murine ovarian cancer [15], and even described as a tool for novel cancer treatment strategy.

In the present study, we have analyzed the potentials and limitations of low abundant-expressed molecules, i.e., ZP2, as cancer biomarkers. For that purpose, real-time PCR analyses were performed with a defined number of gene-specific molecular probes to get an estimate of the probability and sensitivity of such a method. Obviously, when the total number of target molecules to be detected falls below a certain cut-off value, they cannot be detected in each, but only in a portion of distinct PCR-reactions using partitions of one and the same starting material. Thus, a basic question to be addressed for each potential biomarker expressed at a low level is if the experimental effort in terms of time and costs is legitimated by the sensitivity and specificity of the test system. We demonstrate that ZP2 mRNA is expressed in a low-abundant manner with high specificity in (1) subsets of cancer cell lines representing different cancer subtypes and (2) a significant proportion of primary colon cancers. The potential benefit of ZP2 as a biomarker is discussed. In the second part of our study, the function of ZP2 in cancerogenesis has also been analyzed. Since ZP2 shows an enhanced transcript level in colon cancer cells, siRNA experiments have been performed to investigate whether down-regulation of ZP2 would affect cell proliferation. In addition, key molecules involved in proliferation processes, like the cell cycle regulator cyclin D1 [16] and extracellular signal-regulated kinases (ERK1/2) [17], have been studied for their contribution to ZP2 signaling.

## 2. Materials and Methods

### 2.1. Cell Lines

Human tumor cell lines were cultured in DMEM/10% FCS under a humidified 5% CO_2_ atmosphere at 37 °C. The following cell lines were used: A-172, A549, A64-CLS, Hela, HL-60, LNCaP, MCF-7, MG-63, PC-3, SaOs, and SW-480 from Cell Lines Service (Eppelheim, Germany); B-CPAP, BHY, CAL-62, COLO320HSR, H1184, H146, HT29, LoVo, SKN-MC, SW-403, and THP-1 from DSMZ (Braunschweig, Germany); FTC-133, U251-MG, and U373-MG from Sigma-Aldrich (Taufkirchen, Germany); and SW948 and U87-MG from American Type Culture Collection (Manassas, VA, USA), as previously described [18].

### 2.2. Tumor Tissue Samples

Snap frozen (liquid nitrogen) and stored (−80 °C) primary colon cancer tissues and corresponding intraindividual matched control colon tissues were obtained from the Biobank of the University Hospital Bonn according to an approval (#067/18) of the ethical board of the University of Bonn. A number of 30 samples (44%) were from female patients, and 39 samples (56%) were from male patients (see Figure S1).

### 2.3. Quantitative Reverse Transcription-PCR (qPCR)

RNA isolation and RT-PCR analysis (standard conditions): Cells were grown in 6 cm Petri dishes to about 50% confluency, and total cellular RNA was then isolated with the RNeasy^®^ Mini Plus Kit (Qiagen, Hilden, Germany) as described in the manufacturer’s instructions. Then, 1 µg of total cellular RNA was reverse transcribed with the iScript^™^ cDNA synthesis kit according to the manufacturer’s protocol (Bio-Rad Laboratories, Munich, Germany). For the isolation of total RNA from colon tissue, the RNeasy^®^ Fibrous Tissue Mini Kit was used. In brief, 30 mg tissue was homogenized and lysed in RLT buffer using a homogenizer. Then, proteinase K solution was added to the lysate followed by an incubation step for 10 min at 55 °C. After transferring the proteinase K digested lysate to the RNeasy^®^ spin columns, an additional on-column DNA digestion was performed to remove potential DNA contaminations. Finally, total RNA was eluted from spin column, quantified using a NanoDropHND-1000 spectrophotometer (NanoDrop Technologies, Wilmington, DE, USA) and stored at −80 °C. For real-time PCR, the CFX Connect^™^ Real-Time PCR System (Bio-Rad Laboratories, Munich, Germany), SYBR^®^ Green (Bio-Rad Laboratories, Munich, Germany), and gene-specific primers were used. Real-time PCR was performed by adding 50 ng cDNA to a master mix containing primers and iQ™ SYBR^®^ Green Supermix (Bio-Rad Laboratories, Munich, Germany). PCR conditions were as follows: a 5 min preceding denaturation step at 95 °C was succeeded by 50 cycles of 15 s at 95 °C, 30 s at annealing temperatures (AT) specific for the primers, and 30 s at 72 °C for elongation. Relative differential gene expression was calculated using the ΔΔCt-method described by Pfaffl [19] with β-2-microglobulin (B2M) serving as housekeeping gene. The sequences, annealing temperatures, and efficiencies of gene specific primers (Metabion, Martinsried, Germany) were as follows: β-actin (sense 5′-CATGGATGATGATATCGCCGCG-3′, antisense 5′-ACATGATCTGGGTCATCTTCTCG-3′, annealing temperature (AT) = 69 °C, efficiency (E) = 1.94), β-2-microglobulin (B2M) (sense 5′-GCCTTAGCTGTGCTCGCGCT-3′, antisense 5′-TGCTGCTTACATGTCTCGATCCCA-3′; AT = 64 °C; E = 2.04), cyclin D1 (sense 5′-AGCTCCTGTGCTGCGAAGTGGAA-3′, antisense 5′- AGTGTTCAATGAAATCGTGCGGGG-3′; AT = 69 °C; E = 2.07), exosome component 5 (EXOSC5) (sense 5′-CCGGCACTTTGCCTGCGAAC-3′, antisense 5′-GAAGAGAGCCCGCATGGGCA-3′; AT = 67 °C; E = 2.01), glyceraldehyde-phosphate dehydrogenase (GAPDH) (sense 5´-TGGTATCGTGGAAGGACTCA-3′, antisense 5′-CCAGTAGAGGCAGGGATGAT-3′; AT = 67 °C; E = 1.93), peptidylprolyl isomerase A (PPIA) (sense 5′-ACGCCACCGCCGAGGAAAAC-3′, antisense 5′-TCTGCAAACAGCTCAAAGGAGACG-3′; AT = 64 °C; E = 1.98), Ki67 (sense 5′-AAATTCAGACTCCATGTGCCTGAG-3′, antisense 5′-TCAAATATCTTCACTGTCCCTATGAC-3′; AT = 66 °C; E = 1.91), podoplanin (sense 5′-ACAGGTTTGGAAGGCGGCGT-3′, antisense 5′-TCTTGCGCGTGGACTGTGCT-3′; AT = 68 °C; E = 1,97), and ZP2 (sense 5′-GCTCTCTAGCCTGGTCTACTTCCACT-3′, antisense 5′-GTCCATAGCACCTCGTGAGCCA-3′; AT = 58 °C; E = 2.08). PCR products were electrophoresed on 1% agarose gels and visualized with ethidium bromide, isolated, and DNA-sequenced for verification (Figure S2).

### 2.4. Quantification of ZP2 Molecules

For absolute quantification of ZP2 molecules, the human ZP2 Gene ORF cDNA clone vector (Sino Biological, Wayne, NJ, USA) was used.

### 2.5. Small Interfering RNA (siRNA) Experiments

Experiments for knock-down of ZP2 in HT29 cells were carried out using FlexiTube Hs_ZP2_10 (SI04342282) siRNA for ZP2 and AllStars Negative control siRNA from Qiagen. Cell transfection was performed with Lipofectamine^®^ RNAiMAX according to the manufacturer’s protocol with 5 and 10 nM siRNA concentrations. RNA silencing effects were verified by real-time RT-PCR and western blot analysis.

### 2.6. Protein Extraction and Western Blot

HT29 cells were lysed with Cell Lysis Buffer (Cell Signaling Technology, Frankfurt, Germany) containing phosphatase inhibitors and the addition of proteinase inhibitors Roche Complete Mini ULTRA mix (Roche, Mannheim, Germany) for 20 min on ice for isolation of total cellular protein. The extract was spun down in a microcentrifuge (5000 rpm, 4 °C, 5 min), and the supernatant, referred as total cellular protein, was stored at −80 °C. Integral membrane and membrane-associated protein fraction as well as cytosolic protein fraction were separated from HT29 cells using the Mem-PER^TM^ Plus Membrane Protein Extraction Kit (ThermoFisher, Dreieich, Germany) as recommended by the manufacturer. Protein concentrations were quantified using the Pierce^TM^ BCA Protein Assay Kit (ThermoFisher, Dreieich, Germany). Proteins were separated by SDS-PAGE and subsequently electrophoretically transferred onto a PVDF membrane (0.45 µm) (Millipore, Darmstadt, Germany). Successful transfer was verified by staining the membrane with a Ponceau S rouge solution (Serva, Heidelberg, Germany). Each 30 µg of total cellular protein, cytosolic protein fraction, or membrane protein fraction was loaded per lane on polyacrylamide gels. The membrane was destained with water, followed by blocking unspecific binding sites with Tris-buffered saline (TBS) including 0.1% Tween-20, TBST (Bio-Rad Laboratories), and 5% bovine serum albumin, BSA (Sigma-Aldrich), for one hour at room temperature (RT). The following primary antibodies were used: rabbit IgG anti-phospho-ERK1/2 (dilution of 1:1000; #4370), rabbit IgG anti-ERK1/2 (dilution of 1:1000; #4695), rabbit IgG anti-cyclin D1 (dilution of 1:1000; #2978), rabbit IgG anti-ZP2 (dilution 1:200), and rabbit IgG anti-vinculin (dilution of 1:1000; #13901). All antibodies were from Cell Signaling Technology, except for rabbit IgG anti-ZP2 (sc-30222, Santa Cruz Biotechnology, Heidelberg, Germany) and rabbit IgG anti-epidermal growth factor receptor (EGFR) (dilution 1:200; sc-03). The membrane was incubated with the primary antibody overnight at 4 °C in TBST with 5% BSA followed by three washing steps for 20 min at RT in TBST. Horse radish peroxidase (HRP)-conjugated secondary IgG anti-rabbit antibody (Cell Signaling Technology) was diluted in TBST containing 5% BSA. The membrane was incubated for 1 h at RT. After three washing steps for 20 min with TBST, the membrane was incubated with a chemiluminescent substrate for visualization. Detection was carried out using the “SuperSignal^®^ West Femto Maximum Sensitivity Substrate” or “Pierce^®^ ECL Plus Western Blotting Substrate” (Thermo Scientific, Bonn, Germany). Corresponding protein bands were visualized using the ChemiDoc XRS system and analyzed with the “Quantity One^®^” software (Bio-Rad Laboratories, Munich, Germany). Vinculin served as internal standard for cytoplasmic fractions, and EGFR for membrane fractions.

### 2.7. Immunohistochemistry

For immunohistochemistry, formalin fixed and in paraffin embedded primary colon cancer and matched healthy colon tissues were sliced in sections of 2 µm thickness, mounted on glass slides, and dried at 37 °C overnight. Then, tissue slices were deparaffinized, rehydrated, and rinsed with TBS. Endogenous peroxidase was blocked in a methanol/H_2_O_2_ solution for 10 min. The following primary antibodies and dilutions were used for IHC staining: α-EXOSC5 (abx302857; Abbexa Ltd., Cambridge, UK), α-Ki67 (1:400; #9027; Cell Signaling Technologies, Frankfurt, Germany), α-Podoplanin (1:50; sc-134482; Santa Cruz Biotechnology, Heidelberg, Germany), and α-ZP2 (1:50; sc-30222; Santa Cruz Biotechnology, Heidelberg, Germany). For detection of Ki67 in tissue slices, an additional antigen retrieval step was performed by heating slides in a microwave submersed in 0.01 M sodium citrate buffer (pH 6.0). Unspecific binding sites were blocked in all tissue slices with 20% goat serum in TBS for 30 min at RT. All antibodies were incubated in a humid chamber at 4 °C overnight with the exception of α-Podoplanin antibody (incubation time of 1 h at room temperature). Antigen–antibody binding was visualized using the EnVision Detection System Peroxidase/DAB from Dako (Hamburg, Germany) for EXOSC5, Podoplanin, and ZP2, whereas Signal Stain Boost Detection Reagent/Signal Stain DAB was used for detection of Ki67. Finally, Mayer’s Hematoxylin (Merck Eurolab) was used for counterstaining, and the coverslips were mounted in DePeX mounting medium (SERVA Electrophoresis). Photomicrographs were taken with Axioskop 2 (Carl Zeiss, Jena, Germany) equipped with a 20× objective, and images were acquired with an AxioCam MRc camera (Carl Zeiss, Jena, Germany) and AxioVision 4.7 software (Carl Zeiss, Jena, Germany).

### 2.8. Cell Proliferation Assay

To quantify proliferation of HT29 cells after ZP2 knock-down, crystal violet assays were performed. In brief, HT29 cells were seeded in 24-well plates (*n* = 6) and transfected with 10 nM ZP2 siRNA or 10 nM control siRNA for 48 h. Then, cells were washed with phosphate-buffered saline (PBS), fixed with 4% paraformaldehyde (PFA) for 30 min at RT, stained for 1 h with 0.05% crystal violet in distilled water, washed twice with distilled water, and air-dried. Finally, 450 μL methanol was added per well to elute bounded crystal violet, and optical densities at 540 nm were measured.

### 2.9. Statistics

For statistical analysis, the one-way-ANOVA and the posthoc Tukey’s multiple comparison test were carried out with GraphPad Prism 6 Software (San Diego, CA, USA). *p*-values < 0.05 were considered to be statistically significant.

## 3. Results

### 3.1. ZP2 Is Expressed in Subsets of Human Tumor Cell Lines

**Figure 1:**
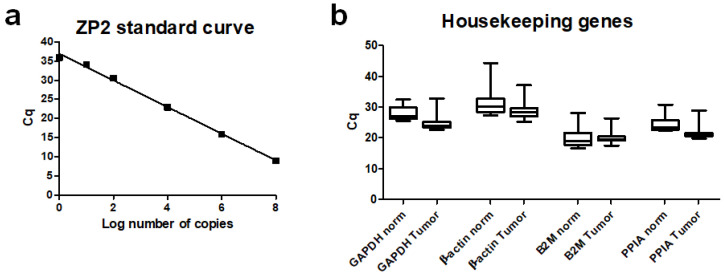
Optimization for qPCR to reliably detect low expression of ZP2. (**a**) Standard curve for ZP2 expression using dilution series with known copy numbers of ZP2-carrying vector molecules. E = 94%; R^2^ = 0.997; slope = −3.48. (**b**) Ct values of housekeeping genes from normal and tumor tissue samples. B2M shows nearly identical expression levels in both tissues.

In order to examine low transcript levels, it is crucial to first optimize the conditions for detection. To figure out the detection limit for real-time (RT)-PCR technology used in the present studies, we performed PCR experiments with a defined number of ZP2-carrying vector molecules. As outlined in Figure 1a, we were able to detect a single molecule per reaction (cycle of threshold (Ct) = 36). Thus, to set a cut-off value of 34 cycles for a reliable ZP2 expression seems to be a plausible assumption, as there the PCR-efficiency is still in a linear range (Figure 1a).

We then analyzed a panel of 28 human tumor cell lines of various origins (Table 1).

Based on methodical reasons, 50 ng cDNA were used per real-time PCR reaction, which corresponds to one twentieth of a total cDNA preparation, as otherwise inhibitory side effects took place. Taking the cell culture conditions into account, we have routinely used 50 ng cDNA that corresponds to about 500 cells. When this type of experiment was repeated four times, the results suggested that in most cell lines, ZP2 transcripts are present only in low amounts, i.e., ZP2 was not detectable in each of the four independent experiments. These data are summarized in Table 1 and show that a consistent expression (four out of four experiments) of ZP2 was detectable only in two cell lines (7%), i.e., colon carcinoma HT29 and SW-403 cells. A 75% probability of ZP2 expression (three out of four experiments) was present in colon carcinoma COLO 320HSR and SW-480 cells, a 50% probability (two out of four experiments) took place in colon carcinoma SW-948 and U251 glioma cells, and a 25% probability was present for twelve cell lines (43%). In seventeen cell lines (61%), ZP2 transcripts were undetectable in all four experiments. An increased probability in expression was strictly correlated with a lower Ct-value. The two cell lines with a 100% probability of ZP2 expression, i.e., HT29 and SW-403 cells, have in common that all four Ct-values (four out of four) were <32. In cell lines with a 75%, 50%, and a 25% probability of ZP2 expression, the average Ct-values were 35 ≥ Ct ≥ 33. Thus, a reliable and reproducible detectability of ZP2 expression relies on a Ct-value ≤ 34. Irrespective of these considerations, a higher ZP2 expression was measured in colon carcinoma cell lines.

### 3.2. ZP2 Is Expressed in Subsets of Primary Colon Cancer Tissues in a Low Abundant Manner

The relatively high level of ZP2 mRNA in colon carcinoma cells prompted us to analyze ZP2 expression in primary colon cancer tissues. A total of 69 primary tissues were analyzed (see Table 2).

**Figure 2:**
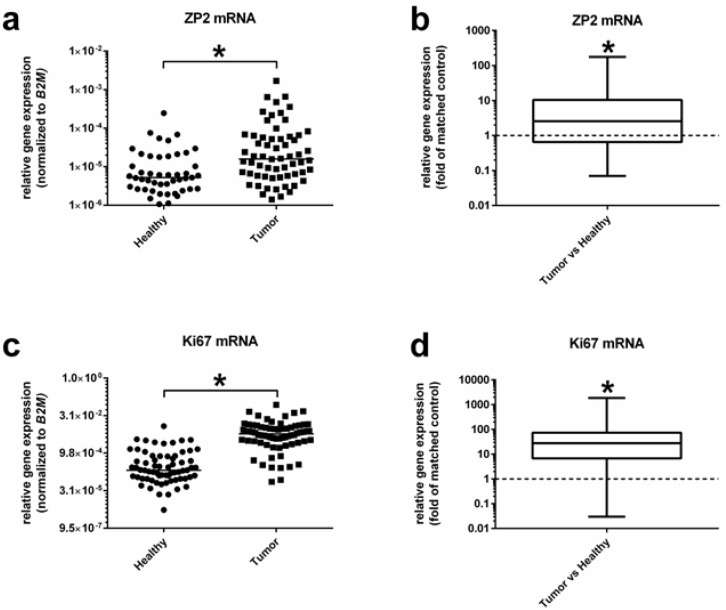
Expression levels of ZP2 and Ki67 in healthy and tumor tissue samples. (**a**) Relative gene expression of ZP2 compared to the housekeeping gene B2M. ZP2 is significantly more highly expressed in tumor cells. (**b**) Differential relative expression of ZP2 compared to matched controls. (**c**) Relative gene expression of Ki67 compared to the housekeeping gene B2M. Ki67 also shows a significantly increased expression in tumor cells. (**d**) Differential relative expression of Ki67 compared to matched controls. Significant differences are depicted with asterisks (* for *p* < 0.05).

To obtain an optimal standardization, we first compared the expression level of the four common housekeeping genes GAPDH, β-actin, B2M, and PPIA in twenty selected cancer and normal colon tissue samples. Whereas the Ct-values between cancer and normal colon tissues varied by about three cycles for GAPDH, β-actin, and PPIA, they were almost identical for B2M (20.20 vs. 20.04) (Figure 1b). Thus, B2M was chosen for all further analyses as the housekeeping gene of choice. To include the quality of the preceding cDNA-synthesis in the real-time PCR reactions, only measurements of samples with a Ct-value of ≤21.0 for B2M were included into data analyses. Table 2 shows that 61 out of 69 tumor samples were ZP2-positive, giving a sensitivity of 88.4% with a statistically significant higher expression rate of threefold of matched controls (Figure 2a,b).

**Figure 3:**
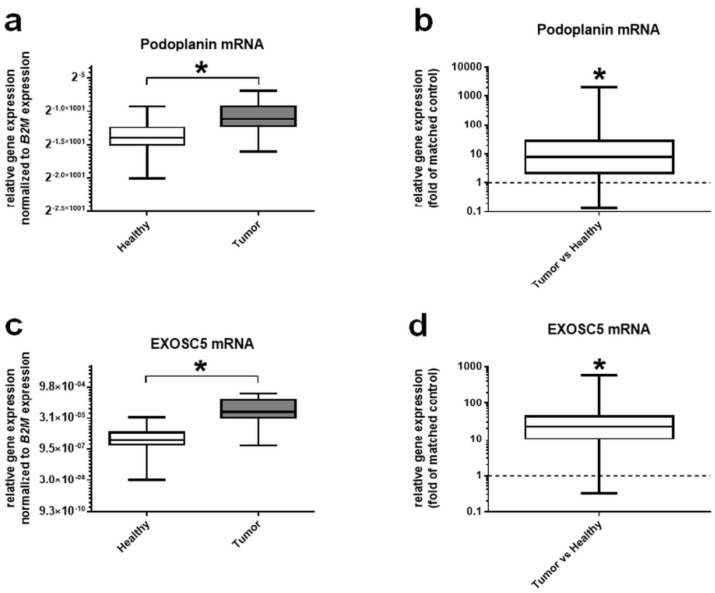
Expression levels of the colorectal cancer marker genes podoplanin and EXOSC5 in healthy and tumor tissue samples. (**a**) Relative gene expression of podoplanin compared to the housekeeping gene B2M. Podoplanin is shown to be significantly more highly expressed in tumor cells. (**b**) Differential relative expression of podoplanin compared to matched controls. (**c**) Relative gene expression of EXOSC5 compared to the housekeeping gene B2M. Additionally, EXOSC5 exhibits a significantly increased expression in tumor cells. (**d**) Differential relative expression of EXOSC5 compared to matched controls. Significant differences are depicted with asterisks (* for *p* < 0.05).

Nevertheless, 48 of the healthy specimens also expressed ZP2, although with a threefold lower expression level (Figure 2a,b), leading to a specificity of 30.4%. To verify the quality of tumor samples, expression of Ki67 as a prominent proliferation marker was analyzed and compared to normal and matched controls. As expected, tumor samples showed an increased Ki67 transcript level of more than 30-fold compared to healthy tissues (Figure 2c,d). Two prognostic colon cancer biomarkers, namely podoplanin [20] and exosome component 5 (EXOSC5) [21], have been chosen to further verify tumor and normal tissue specimens: podoplanin mRNA level was significantly increased by 10-fold in tumor samples (Figure 3a,b), and EXOSC5 by even 20-fold (Figure 3c,d). Additional data summarizing the specificity of ZP2 analysis are shown in Figure S3.

**Figure 4:**
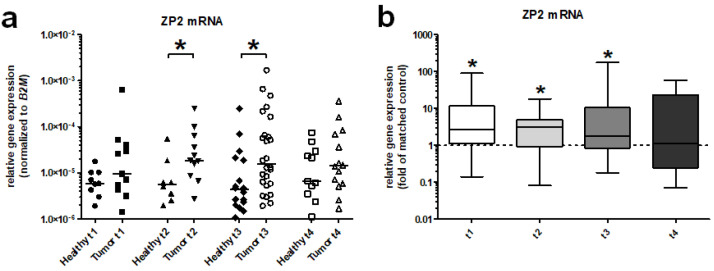
Differential relative gene expression analysis of ZP2 in different tumor stages. (**a**) Comparison of ZP2 expression levels between different tumor stages (T1–T4) with their healthy matched control tissue samples. ZP2 exhibits a significantly increased expression in T2 and T3 stage tumor specimens vs. controls. (**b**) Differential relative gene expression analysis of ZP2 focusing on different tumor stages, which shows significantly increased levels of transcription in T1, T2, and T3 compared to their matched healthy controls. Significant differences are marked with asterisks (* for *p* < 0.05).

In addition, a more detailed differential analysis focusing on different tumor stages revealed a significantly higher ZP2 expression rate in T2 and T3 tumor stages (Figure 4a). When compared to matched controls, even T1 stage tumors exhibited a significantly increased ZP2 expression (Figure 4b). Details of clinical data from all patients examined are listed in Figure S1.

### 3.3. Immunohistochemical Analysis of ZP2 in Colon Cancer Tissue

**Figure 5:**
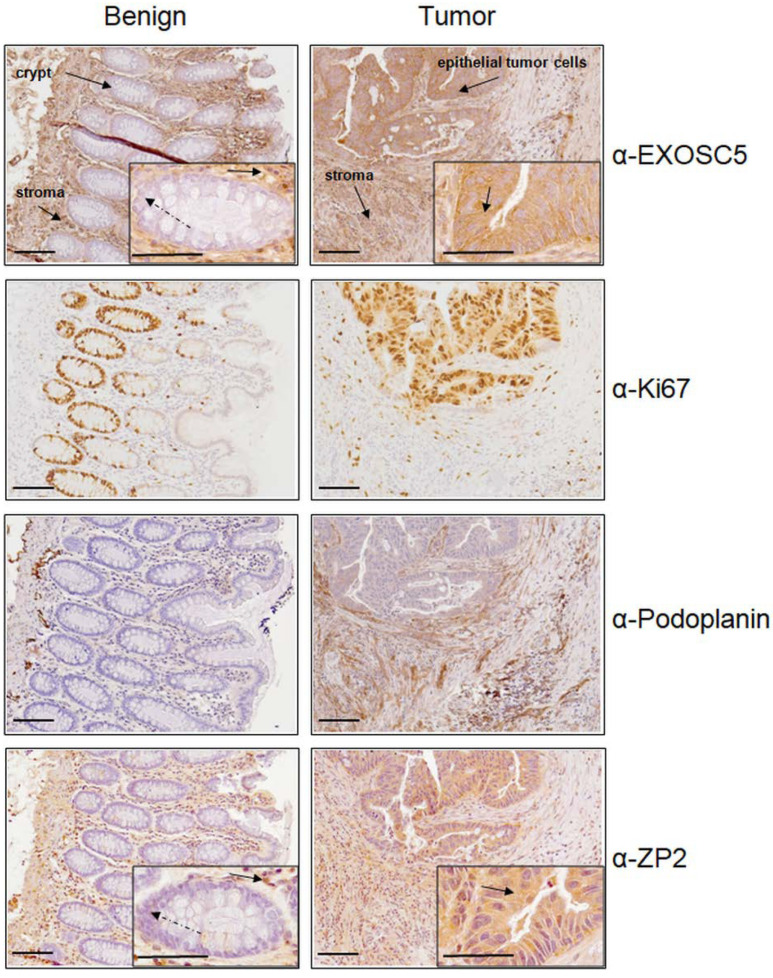
Representative EXOSC5-, Ki67-, podoplanin-, and ZP2-immunostaining in human tissues. On the left, case-wise matched benign colon mucosa, the right panel shows consecutive sections of the corresponding colon carcinomas (all 200×, bar represents 100 µm). Antigen-positive cells are stained in brown; antigen-negative cells are counterstained in blue. Insets with a higher magnification show antigen-positive cells marked with a black arrow and antigen-negative cells marked with a dashed arrow (all 400×, bar represents 100 µm).

In order to investigate whether ZP2, Ki67, podoplanin, and EXOSC5 share the same tissue distribution, immunohistological analysis of colon cancer specimens has been performed (Figure 5).

In benign mucosa, EXOSC5 is seen in stromal cells, while cryptal epithilium remains largely unstained. In contrast, epithelial tumor cells show a strong immunereactivity, as does the tumor stroma. Ki67 labels the basal zone of cryptal epithelium and shows increased numbers of proliferating cells in malignant tumors in a haphazard fashion. Podoplanin stains endothial cells in benign and malignant colon tissues; however, in malignant tissues, an additional myofibroblastic staining was observed. ZP2 is widely expressed in the stroma of benign and malignant colon tissue; additionally, it is detected in epithelial tumor cells. Notably, cryptal epithelia are unstained, too. The tissue distributions of EXOSC5 and ZP2 are conspicuously similar, in benign and malignant colon tissues alike.

### 3.4. ZP2 Promotes Colon Cancer Cell Proliferation

**Figure 6:**
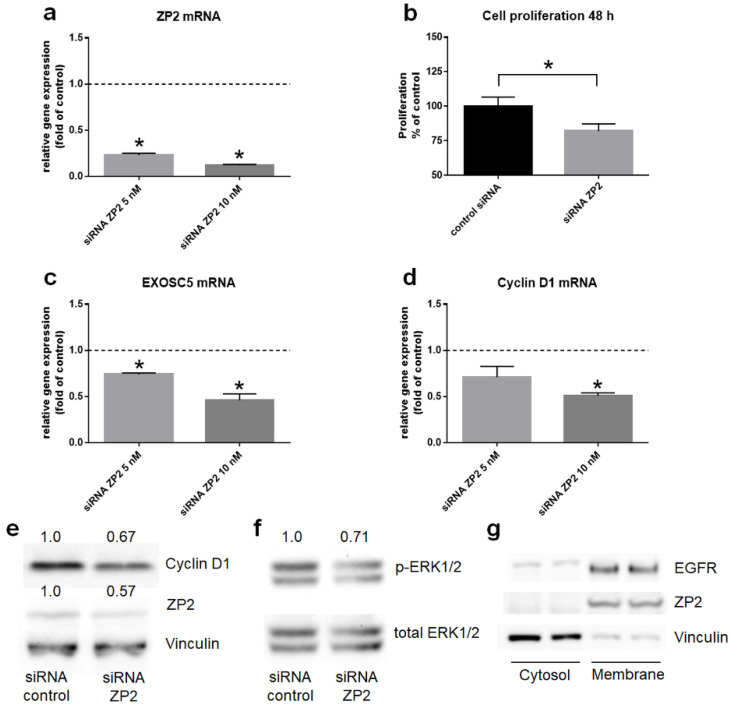
ZP2 silencing effects on colon tumor HT29 cells. (**a**) Decreased expression level of ZP2 after 48 h treatment with its siRNA compared to control siRNA. Untreated control showed no difference compared to control siRNA. Statistically significant differences to control with *p* < 0.05 are shown with asterisks (*). (**b**) Reduction of cell proliferation rate after treatment with 10 nM siRNA for ZP2 compared to non-stimulated cells as control (= 100% at y-axis). Statistically significant difference with *p* < 0.05 is shown with an asterisk (*). (**c**) Decreased EXOSC5 mRNA level after incubation with siRNA for ZP2 compared to control siRNA. Statistical significant differences (*p* < 0.05) compared to control are designated with asterisks (*). (**d**) Reduced cyclin D1 transcript level after incubation with siRNA for ZP2 compared to control siRNA. Asterisk (*) shows statistically significant difference (*p* < 0.05) to control. (**e**) Western blot analysis for cyclin D1 and ZP2 after treatment of HT29 cells with siRNA for ZP2. Vinculin served as internal standard. Numbers show fold change of signal compared to control siRNA (set as “1.0”). Values are means from two independent experiments with standard deviations for cyclin D1 (±0.15) and for ZP2 (±0.11). (**f**) Western blot analysis for phospho-ERK1/2 (p-ERK1/2) after incubation with siRNA for ZP2 compared to total ERK1/2. Numbers show fold change compared to control siRNA (set as “1.0”). Value is mean from two independent experiments with standard deviation (±0.13). (**g**) Western blot analysis for cellular distribution of ZP2, which is highly enriched in the membrane fraction, while the cytosolic marker protein vinculin is mainly detected in the cytosol fraction. EGFR served as the marker for membrane fractions. The fractions were from the same lysate with the same amount of protein (30 µg) loaded on the gel.

Since ZP2 shows an enhanced transcript level in colon cancer tissue, it seemed obvious to investigate its potential function in proliferative cellular processes. Hence, the colon tumor cell line with the highest ZP2 expression rate, namely HT29 (Table 1), has been chosen for studying ZP2 effects on proliferation. For this purpose, HT29 cells were treated with 10 nM ZP2-specific siRNA for 48 h, which led to a reduction of ZP2 mRNA and protein level compared to the control (Figure 6a,e). As a consequence, the cell proliferation rate decreased (Figure 6b), as well as the mRNA level of EXOSC5 (Figure 6c), the transcript and protein level of cyclin D1 (Figure 6d,e), and also the phosphorylation state of ERK1/2 (Figure 6f). Since the intracellular kinases ERK1/2 have been shown to be affected by ZP2 silencing, the question arose of whether ZP2 could be a potential upstream target molecule, like membrane-bound receptors. Thus, it was examined if ZP2 is located in the cell membrane of HT29 cells, which could indeed be demonstrated by western blot analysis (Figure 6g). Additional original data are shown in Figures S4–S6. 

## 4. Discussion

Our study shows for the first time the expression of the zona pellucida glycoprotein ZP2 in various tumor cell lines as well as in tissue samples of healthy and cancerous colon. To our knowledge, only one publication has focused on the expression of ZP glycoproteins in human tumors. Inter alia, Costa and colleagues [11] have demonstrated that ZP1, ZP3, and ZP2, but not ZP2-specific transcripts, are expressed in prostate cancer-derived PC3 cells and prostate tumor tissue. Surprisingly, the authors demonstrated ZP1 to ZP4 expression on the protein level, which points to differences in the specificity and/or sensitivity of the methods applied. In our study, we have basically concentrated on the potential of ZP2 expression as a biomarker tool for risk estimation in cancer diagnosis. For that purpose, various tumor cell lines and more than 60 tissue samples from colon cancer patients were examined to analyze the potential of ZP2 as a putative diagnostic tumor marker. In contrast, ZP1, ZP3, and ZP4 were not detectable at all or only in a small number of colon cancer specimens (Figure S3). Ovary tissue samples were used as positive controls, although showing undetectable amounts for ZP4 exprression (Figure S7).

The data we have provided for the expression of ZP2 in human malignant cell lines can be seen as an example of the so-called Monte Carlo effect that has been described as an intrinsic limitation of quantitative PCR raised by the fact that below a certain copy number, the PCR reaction generates a detectable number of products in a non-reproducible fashion [22,23].

We would like to suggest that the expression pattern of genes such as ZP2 in cancer is linked to a general (epi)genetic instability inherent to such malignancies that inter alia leads to an increased “background noise” of low-abundant gene expression [24]. Such an augmented background noise can lead to a heightened probability in the expression level of low abundant genes, which is reflected in an increase in the number of positive PCR reactions (in comparison to normal cells or tissues) performed with the same partitioned sample (derived from one specific subculture of one specific cell line) or with one aliquot taken from different samples that are derived from various subcultures of the same cell line.

Thus, the expression pattern of ZP2 in tumor cells may provide an opportunity for the detection of circulating or disseminated tumor cells in body fluids, and, in principle, for an early diagnosis of cancer via a negative selection mode strategy [25,26]. Apart from the analysis of circulating tumor cells, also tumor-derived exosomes could be an object of investigation [27]. The efficiency of such methods could be increased when combined with the search for circulating tumor DNA [28,29].

Basic questions that need to be answered to obtain a frame on the reliability of early cancer detection include: (i) at which stage do developing tumors release cells, exosomes, or DNA into the blood stream or into other body fluids such as urine or saliva? (ii) What is the number of cells or molecules that are released from tumors and, based on these data, what is the minimal necessary volume of body fluids that has to be analyzed?

It also has to be kept in mind that ZP2 represents only one candidate of low-abundant expressed genes that are almost exclusively found in tumor cells. We have also simultaneously examined two well-known biomarkers for colon cancer, podolanin [20] and EXOSC5 [21], to better differentiate between healthy and cancerous tissues. Podoplanin and EXOSC5 were shown to be more highly expressed in colon cancer as well [20,21], although to a minor extent compared to our study, which could be due to a lower number of tissue samples.

These results suggest that specific sets of low abundant expressed cancer-specific genes should be analyzed in parallel to obtain the most useful combination for tumor diagnosis in various cancers.

**Figure 7:**
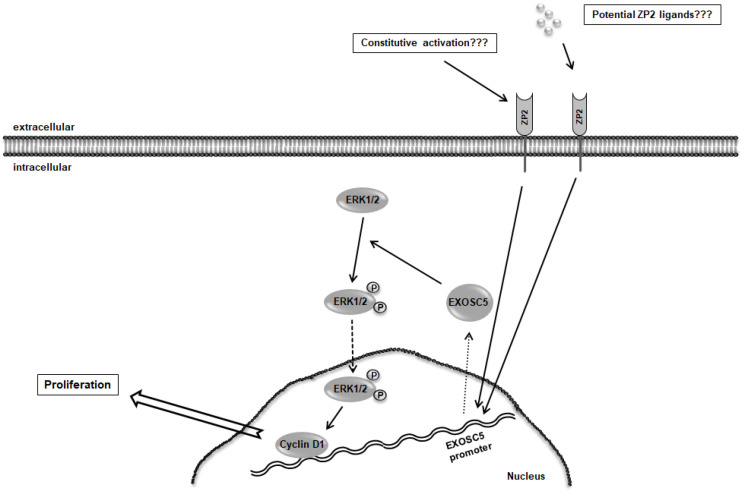
Schematic summary shows the proposed function of ZP2 in participating in colon cancer cell proliferation. It is speculated that ZP2 might act as a membrane-bound receptor-like protein, which could positively affect EXOSC5 expression, leading to ERK1/2 activation and thus up-regulation of cyclin D1, hence showing a potential impact on cell proliferation. Black arrows represent activating processes, the dashed arrow shows exclusive transport, the dotted arrow shows increased biosynthesis as well as transport, while the block arrow exhibits a cellular process (proliferation).

After having explored that ZP2 exhibits an enhanced mRNA level in colon cancer cells, the next step was to investigate its putative function in cell proliferation. For this purpose, ZP2 mRNA and protein levels were reduced by RNA silencing experiments. Successful down-regulation of ZP2 gene products have led to a decreased cell proliferation rate in connection with a decreased mRNA level of EXOSC5, protein level of the cell cycle regulator cyclin D1, and also of phospho-ERK1/2 state. Both proteins are key regulators in the G1- to S-phase transition [16,17]. Activation of ERK1/2 leads to induction of cyclin D1, which in consequence is necessary for the entry into the S-phase of the cell cycle [17]. The same pathways have been identified to be regulated by EXOSC5 in colon cancer as well [21]. Hence, ZP2 seems to be affecting the cell proliferation rate upstream via the EXOSC5-ERK1/2-cyclinD1-pathway. According to our data, we propose that in colon tumor cells, ZP2 is expressed as a plasma membrane protein that, thereby, is capable as an upstream receptor and regulator of driving EXOSC5-ERK1/2-cyclinD1-signaling (Figure 7).

To verify our hypothesis, it is necessary to prove that ZP2 and EXOSC5 are expressed in the same cells. Immunohistological analysis of colon tissue has shown that this seems to be the case (Figure 5). EXOSC5 and ZP2 have been found in the same tissue structure in benign and also cancerous colon tissue, which supports our hypothesis. Although Ki67 is shown to not be essential for proliferation, it is, however, a marker of cell-cycle entry with its highest levels in G_2_ and M-phase. Senescent or quiescent cells do not express detectable Ki67 levels [30]. Additionally, Ki67 expression is correlated to a decrease of tissue differentiation [31]. Hence, Ki67 can be used as marker for cancerous cells. The expression pattern we have observed for podoplanin, i.e., in stromal fibroblasts in colon cancer tissue and endothelial cells of lymphatic vessels in benign colon tissue, is in agreement with previous studies [20,32].

It has recently been described that murine mature transmembrane-bound ZP2 does not interact with partners in the cell membrane of oocytes [10], and thus may act as a single receptor molecule. SED1 (a secreted protein that contains notch-like epidermal growth factor (EGF) repeats and a discoidin/FS/8 type C-domain) has been proposed to be a ZP2 sperm receptor candidate [33]. The fact that EGF-like domains bind to ZP2 may give some hints of putative ligands, e.g., EGF or structure analogs [34], which could be responsible for activating pro-proliferative cell processes. Furthermore, the fact that ZP2 is located in the plasma/cytoplasmic membrane makes it a potential target molecule for tumor therapy. It has been reported earlier that immunization of mice against ZP3 prevented ovarian tumorigenesis [15]. A similar approach could also be useful in respect to colon cancer with antibodies against ZP2.

## 5. Conclusions

In summary, our study shows ZP2 to be a new biomarker for diagnosis, best used in combination with other low abundant tumor marker genes in colon cancer. Furthermore, ZP2 promotes cell proliferation via the ERK1/2-cyclin D1-signaling pathway. In future, it seems warranted to search for potential ligands of membrane-bound ZP2 that interfere with cancer-related processes, such as the cell cycle and signaling cascades.

## Figures and Tables

**Table 1:** Expression of ZP2 transcripts in human tumor cell lines as revealed by real-time PCR. Four separate RT-PCR experiments were carried out with the corresponding individual Ct-Values (Ct1 to Ct4). Percentage of expression detected in the analyzed individual samples (Ct ≤ 36) are shown in the right column (grey box with value > 0). Not detectable (Ct ≥ 37) is designated as “Nd”. Colon carcinoma cell lines are given in bold.

Cell line	Origin	C_t1_	C_t2_	C_t3_	C_t4_	%
A-172	Glioblastoma	nd	nd	nd	nd	0
A549	Non-small cell lung carcinoma	34.4	nd	nd	nd	25
A64-CLS	Submaxillary gland adenoma	nd	nd	nd	nd	0
B-CPAP	Papillary thyroid carcinoma	nd	nd	nd	nd	0
BHY	Oral squamous cell carcinoma	nd	nd	nd	nd	0
CAL-62	Thyroid anaplastic carcinoma	34.5	nd	nd	nd	25
**COLO 320HSR**	**Colon carcinoma**	**34.3**	**33.9**	**nd**	**35.0**	**75**
FTC-133	Follicular thyroid carcinoma	nd	nd	nd	nd	0
H1184	Small cell lung carcinoma	nd	nd	nd	nd	0
H146	Small lung cell carcinoma	35.0	nd	nd	nd	25
HeLa	Cervix adenocarcinoma	nd	nd	nd	nd	0
HL-60	Leukemia (promyelocytic)	nd	nd	nd	nd	0
HN	Oral squamous cell carcinoma	34.9	nd	nd	nd	25
**HT-29**	**Colon carcinoma**	**31.0**	**31.9**	**31.2**	**31.8**	**100**
LNCaP	Prostate carcinoma	nd	nd	nd	nd	0
**LoVo**	**Colon carcinoma**	**nd**	**nd**	**34.5**	**nd**	**25**
MCF-7	Breast carcinoma	nd	nd	nd	nd	0
MG-63	Osteosarcoma	34.6	nd	nd	nd	25
PC-3	Prostate adenocarcinoma	35.0	nd	nd	nd	25
SaOs	Osteosarcoma	nd	nd	nd	nd	0
SKN-MC	Neuroblastoma	nd	nd	nd	nd	0
**SW-403**	**Colon carcinoma**	**31.4**	**31.9**	**31.7**	**31.9**	**100**
**SW-480**	**Colon carcinoma**	**33.3**	**34.5**	**nd**	**35.0**	**75**
**SW-948**	**Colon carcinoma**	**nd**	**nd**	**34.9**	**34.7**	**50**
THP-1	Leukemia	nd	nd	nd	nd	0
U251-MG	Glioblastoma	35.0	34.7	nd	nd	50
U373-MG	Glioblastoma (astrocytoma)	nd	nd	nd	nd	0
U87-MG	Glioblastoma	34.6	nd	nd	nd	25

**Table 2:** Descriptive analysis of ZP2 expression in human colon cancer and intraindividual matched control tissues as revealed by real-time PCR and agarose gel electrophoresis. Four separate RT-PCR experiments per tissue sample were performed. The data result in a sensitivity of 88% (61 positives out of 69) and a specificity of 30% (48 positives in controls) for ZP2.

Tissue Samples	Healthy	Tumor	∑
positive	48	61	109
negative	21	8	29
∑	69	69	138

## Data Availability

Supporting data and materials are available on request to the corresponding author.

## Supplementary Materials

The following are available online at https://www.mdpi.com/article/10.3390/cancers13081759/s1: Figure S1: Table including sex, age, histopathological tumor staging and grading of patients with colon cancer, Figure S2: (a) Melting curve analysis of qPCR experiments for ZP2 mRNA detection (T_m_ = 85.5 °C); (b) qPCR product of HT29 cells using specific ZP2 primers was sequenced at Eurofins Genomics Germany GmbH, Figure S3: Images of PCR products (a: ZP2 (289 bp); b: ZP3 (375 bp) separated by agarose gel electrophoresis, Figure S4: Uncropped images of Western Blot of HT29 cell lysates in ZP2 siRNA experiments (Ref. Fig. 6e), Figure S5: Uncropped images of Western Blot of HT29 cell lysates in ZP2 siRNA experiments (Ref. Fig. 6f), Figure S6: Uncropped images of Western Blot of HT29 cell lysates in ZP2 siRNA experiments (Ref. Fig. 6g), Figure S7: Expression level of ZP1, ZP2, ZP3 and ZP4 in ovarian RNA designated as Ct-values.
